# Secreted Proteins from the Helminth *Fasciola hepatica* Inhibit the Initiation of Autoreactive T Cell Responses and Prevent Diabetes in the NOD Mouse

**DOI:** 10.1371/journal.pone.0086289

**Published:** 2014-01-21

**Authors:** Maria E. Lund, Bronwyn A. O'Brien, Andrew T. Hutchinson, Mark W. Robinson, Ann M. Simpson, John P. Dalton, Sheila Donnelly

**Affiliations:** 1 School of Medical and Molecular Biosciences, University of Technology Sydney, New South Wales, Australia; 2 School of Biological Sciences, Queen's University Belfast, Belfast, Northern Ireland; 3 Institute of Parasitology, McDonald Campus, McGill University, St. Anne de Bellevue, Quebec, Canada; 4 The i3 Institute, University of Technology Sydney, New South Wales, Australia; Université Paris Descartes, France

## Abstract

Infections with helminth parasites prevent/attenuate auto-inflammatory disease. Here we show that molecules secreted by a helminth parasite could prevent Type 1 Diabetes (T1D) in nonobese diabetic (NOD) mice. When delivered at 4 weeks of age (coincident with the initiation of autoimmunity), the excretory/secretory products of *Fasciola hepatica* (FhES) prevented the onset of T1D, with 84% of mice remaining normoglycaemic and insulitis-free at 30 weeks of age. Disease protection was associated with suppression of IFN-γ secretion from autoreactive T cells and a switch to the production of a regulatory isotype (from IgG2a to IgG1) of autoantibody. Following FhES injection, peritoneal macrophages converted to a regulatory M2 phenotype, characterised by increased expression levels of Ym1, Arg-1, TGFβ and PD-L1. Expression of these M2 genetic markers increased in the pancreatic lymph nodes and the pancreas of FhES-treated mice. *In vitro*, FhES-stimulated M2 macrophages induced the differentiation of Tregs from splenocytes isolated from naïve NOD mice. Collectively, our data shows that FhES contains immune-modulatory molecules that mediate protection from autoimmune diabetes via the induction and maintenance of a regulatory immune environment.

## Introduction

Type 1 Diabetes (T1D) is a multifactorial autoimmune disease in which the insulin-secreting beta (β) cells within the pancreatic islets are destroyed. While disease susceptibility is determined by genetic, immunological and environmental factors, the observed rising incidence of T1D in recent decades suggests a significant etiological role for environmental influences, either the removal of a protective factor(s) or introduction of a susceptibility factor(s) [1]. Post-industrial improvements in sanitation and living conditions have led to a dramatic decline in exposure to pathogens, notably parasitic worms (helminths), among Western populations [2]–[4]. Epidemiological studies have shown that the absence of endemic helminth infection is inversely correlated with the incidence of T1D [5], [6]. This suggests that exposure to helminths represents a predominant protective environmental factor against the development of T1D, and auto-inflammatory diseases in general. It has been proposed that the controlled reintroduction of helminth infection into Western populations could represent an effective therapy for auto-inflammatory diseases [10], [11]. Support for the therapeutic potential of helminth infection in the prevention of autoimmune diabetes has come from experimental studies showing that infection of mice with helminth parasites prevents the development of T1D [12]–[17].

Mammals infected with a helminth parasite exhibit a potent and biased Th2-driven immune response during the acute phase, which counter-regulates Th1-driven autoimmune pathologies [12], [13], [15], [18]. During the chronic phase of infection, however, immune-regulatory networks emerge. These are driven primarily by regulatory T cells (Tregs) producing IL-10 and TGFβ [18], which has the bystander effect of protecting against Th1-associated autoimmune diseases, such as T1D [16]. It is likely that the ability of helminth parasites to modulate host immune responses towards an anti-inflammatory/regulatory phenotype is attributable to the molecules that the parasites secrete and/or excrete which interact with immune effector cells to modulate their function [19], [20].

We have previously reported that the excretory/secretory products of the helminth parasite *Fasciola hepatica*, termed FhES, collected after culturing parasites *in vitro*, exerts a potent immune-modulatory effect in the immunocompetent host (Balb/c and C57BL6 mice). This is achieved by the activation of regulatory M2 macrophages [21], [22], suppression of dendritic cell (DC) maturation [23], and inhibition of antigen-specific Th1 and Th17 cell differentiation [23], [24]. Given these profound immune-modulatory properties, in this study we examined the potential of FhES to prevent the initiation and perpetuation of the autoreactive immune responses that underpin T1D development.

Short-term intra-peritoneal administration of FhES to female non-obese diabetic (NOD) mice resulted in permanent protection against immune-mediated β-cell destruction. Disease prevention was associated with the induction of a regulatory immune environment composed of regulatory B cells (Bregs), and M2 macrophages that induced the differentiation of Tregs. These data support the proposition that helminth-derived molecules may represent a more desirable therapeutic alternative to the use of live parasitic infection as a treatment for T1D, and other auto-inflammatory diseases.

## Materials and Methods

### Ethics statement

Four week old female NOD/Lt mice were purchased from the ARC (Perth, Australia) and maintained under an experimental protocol approved by the University of Technology Sydney (UTS) Animal Care and Ethics Committee (Approval Number: 2010-432A). *F. hepatica* infections in sheep were performed at the approval of the Animal Ethic Committee (AEC) located at the Elizabeth Macarthur Agricultural Institute (EMAI, Menangle, New South Wales (NSW), Australia) and under the guidelines established by the Animal Research Review Panel (ARRP) of the NSW Department of Primary Industries (DPI) (www.animalethics.org.au).

### Preparation of FhES

Mature *Fasciola hepatica* were recovered from the bile ducts of Merino sheep 16 weeks after an experimental infection and FhES was prepared by maintaining the parasites in culture for 8 h as previously described [21]. The culture medium was concentrated to 1 mg/ml, using a 3000 Da cut-off centricon, filter-sterilised and stored at −80°C until required.

### Treatment of NOD mice with FhES

FhES (10 µg in 100 µl sterile PBS) was delivered to mice intraperitoneally on alternate days for a total of 6 injections. Control mice received 100 µl of sterile PBS. Glucose levels were measured from tail vein blood weekly, from 13 weeks of age, using Accu-check Advantage blood glucose strips (Roche, Australia). Animals were sacrificed at diabetes onset; defined by two consecutive blood glucose concentrations above 14 mmol/L. All efforts were made to minimize suffering.

### Scoring of Insulitis

Formalin-fixed paraffin-embedded pancreata were sectioned (4 µm) at three non-overlapping levels, such that each section was separated from the preceding one by at least 20 µm. Sections were stained by hematoxylin and eosin (H&E), studied for their histological characteristics, and graded for insulitis on a scale of 0–4; whereby 0 = healthy islet or mild peri-insular mononuclear cell infiltration, 1 = infiltration up to 25% of islet mass, 2 = infiltration up to 50% of islet mass, 3 = infiltration from 50% up to 75% of islet mass, and 4 = less than 25% of islet mass present. Slides were assessed in a blinded fashion and all islets in 10 slides from each pancreas were scored.

### Characterisation of autoantigen-specific immune responses

The levels of anti-insulin and anti-glutamic acid decarboxylase (GAD) immunoglobulin in sera were determined by ELISA, as previously described [12]. Briefly, plates were coated with bovine insulin (10 µg/ml; Sigma, Australia) and bound antibodies in sera detected by the addition of either goat anti-mouse IgG1, IgG2a (BD Pharmingen, Australia) or IgM (Sigma, Australia) conjugated to alkaline phosphatase. The development of colour after addition of *p*-nitrophenylphosphate (Sigma, Australia) was recorded by spectrophotometry at 405 nm.

For analysis of T cell responses, single cell suspensions were prepared from the spleens of treated mice and cultured (1×10^6^ cells/ml) in the presence of bovine insulin (10 µg/ml; Sigma, NSW, Australia) or anti-CD3 (10 µg/ml; BD Pharmingen, Australia). After 72 h incubation at 37°C, supernatants were collected and analysed for the presence of IL-4 and IFN-γ by ELISA (BD Pharmingen, Australia)

### Characterisation of immune cell populations by flow cytometry

Cells were collected from the peritoneal cavity of treated mice by lavage with 5 ml sterile PBS/BSA1%/heat inactivated FCS2%/0.05% sodium azide. Pancreatic lymph nodes (PLNs) from treated mice were harvested into RPMI (Life Technologies, Australia). Single cell suspensions from both were blocked with anti-CD16/32 mouse Fc Block (BD Pharmingen, Australia) and analysed for the expression of cell surface markers using combinations of the following antibodies: CD3 (SK7), CD4 (L3T4), CD8a (53-6.7), B220 (RA3-6B2), F4/80 (BM8), CD25 (7D4), PD-L1 (M1H5) or CD19 (1D3) (BD Pharmingen or Life Technologies, Australia). For the identification of regulatory T cells, expression of the intracellular marker, Foxp3, was quantified using a mouse Foxp3 intracellular staining kit (BD Pharmingen, Australia). Appropriate isotype control antibodies were used. Labelled cells were analysed using the BD LSRII flow cytometer (BD Biosciences). Data were analysed using FCS Express 4 Cytometry software (De Novo Software). Gating strategies are shown in Fig S1.

### Characterisation of IL-10 secreting cells

A Mouse IL-10 Secretion Assay (Miltenyi Biotec, Australia) was used to identify and quantify the IL-10 secreting cells within the peritoneal cavity and the pancreatic lymph nodes (PLNs) of mice treated with FhES or PBS. In preparation for the assay, single cell suspensions of PLNs harvested from treated mice were cultured overnight in RPMI with 10% v/v heat inactivated FCS (Life Technologies, Australia). Peritoneal cells were harvested by lavage and analysed immediately using the IL-10 secretion assay. Initially, cells were labelled with a capture antibody specific for mouse IL-10, then returned to culture for 45 min at 37°C in RPMI with 10% v/v heat inactivated FCS. Cells were then stained with an IL-10 detection antibody or isotype control antibody, before being counterstained for cell surface markers CD19 or F4/80 to identify B cells and macrophages, respectively. Dead cells were excluded using Dapi staining (Life Technologies, Australia). Labelled cells were analysed using the BD LSRII flow cytometer (BD Biosciences). Data were analysed using FCS Express 4 Cytometry software (De Novo Software).

### Quantification of macrophage-secreted cytokines

Peritoneal macrophages harvested from PBS or FhES-treated mice were cultured overnight in RPMI without any further stimulation. The concentration of IL-10, IL-12 and TGFβ secreted into culture supernatants was measured by ELISA (BD Biosciences and R&D systems)

### Gene expression analysis

Total RNA was extracted from PLNs, peritoneal macrophages or frozen pancreatic tissue using an RNeasy plus mini kit (Qiagen). Gene expression levels of Ym1, Retnla, TGFβ, Arg-1, Foxp3, and β-actin were quantified in real time using Taqman gene expression assays (Applied Biosystems, Australia) and RT-PCR, as previously described [21]. Gene expression was quantified (in triplicate) using the change in cycle threshold method (C_t_^Gene^−C_t_^House Keeping^) and normalised to expression of the house-keeping gene, glyceraldehyde-3-phosphate dehydrogenase. Expression levels of genes in treatment samples were determined by comparison to the average ΔC_t_ of the untreated control cohort.

### *In vitro* macrophage and splenocyte co-cultures

Macrophages were harvested from the peritoneal cavity by lavage and isolated to >94% purity by adherence to plastic for 1 h at 37°C. Splenocytes were cultured (in 96 well flat bottomed plates) with FhES (20 µg/ml), soluble egg antigens (SEA; 50 µg/ml) of the parasitic helminth *Schistosoma mansoni* (Theodor Bilharz Research Institute, Cairo, Egypt.), or autologous peritoneal macrophages (at a ratio of 1∶5), and stimulated with anti-CD3 (2 µg/ml; 17A2; BD Pharmingen, Australia) for 72 h at 37°C in RPMI, supplemented with 10% v/v heat inactivated FCS (Life Technologies, Australia).

### Statistical analysis

Blood glucose data was assessed using survival analysis, and Kaplan-Meier estimates of the survivor functions were compared using a Tyrone-Ware nonparametric test. For insulitis scores, the distributions of scores across mice for each group were determined using a maximum likelihood 8^2^ contingency table test. To compare the overall distributions of scores for the groups a log-linear model was used. Statistical analyses of data for cytokine secretion and immunophenotyping by flow cytometry were performed using the GraphPad Prism 5 for Windows (GraphPad Software Inc.). For comparison of two variables the unpaired Student's t-test with Welch's correction for unequal variances, or the Mann-Whitney two-tailed t test, were used. Error bars represent ± standard error of the mean.

## Results

### Short term peritoneal administration of FhES prevents the onset of T1D

Female NOD mice were injected intraperitoneally with FhES beginning at 4 weeks of age (co-incident with the priming of autoreactive T cell populations and initiation of insulitis) and continuing on alternate days for a total of 6 treatments (10 µg/injection). This treatment regime was chosen to replicate the quantities of FhES that would be secreted during a low-dose infection by *F. hepatica* parasites as they migrate through the peritoneal cavity towards the liver. In addition, we have previously reported that this treatment regime is sufficient to inhibit the development of antigen-specific Th1 immune responses in immune-competent nondiabetes-prone mice [21], [22].

**Figure 1:**
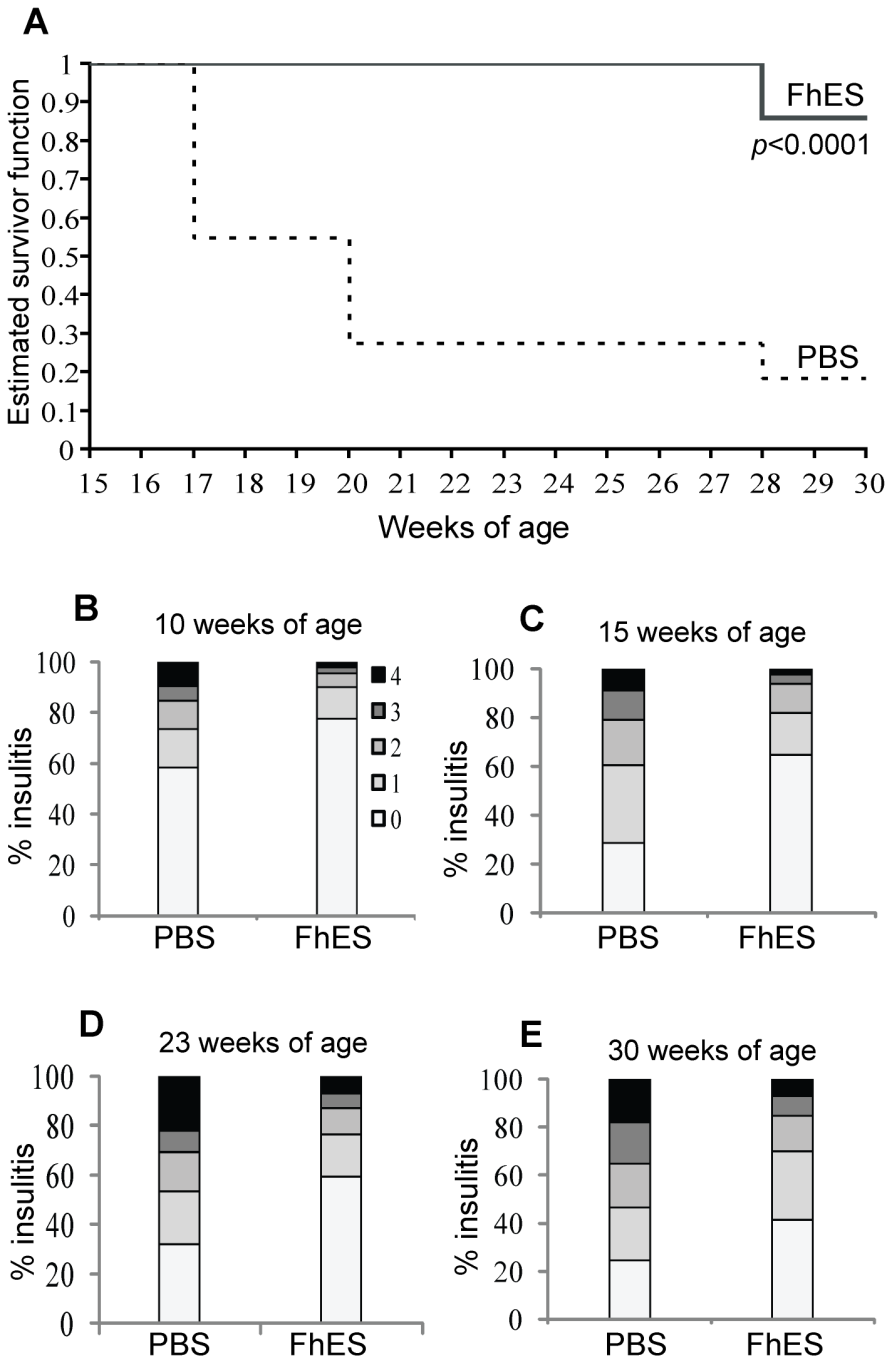
Treatment of NOD mice co-incident with the initiation of autoimmunity prevents T1D. Four-week old female NOD mice were injected intraperitoneally with FhES (10 µg in 100 µl sterile PBS) or vehicle (PBS), on alternate days, for a total of six injections. (A) Blood glucose levels were monitored and animals were sacrificed when they became diabetic (as defined by two consecutive blood glucose concentrations ≥14 mmol/L). The graphs represent an analysis of the age at which each animal was sacrificed and was performed using survival analysis. Data shown is from one of three independent experiments, all of which produced the same outcome. (B–E) Pancreas isolated from mice at 10 (n = 13), 15 (n = 15), 23 (n = 16) and 30 (n = 16) weeks of age were graded for insulitis on a scale of 0–4; whereby 0 = healthy islet or mild peri-insular mononuclear cell infiltration, 1 = infiltration up to 25% of islet mass, 2 = infiltration up to 50% of islet mass, 3 = infiltration from 50% up to 75% of islet mass, and 4 = less than 25% of islet mass present. The proportion of islets with each grade of insulitis is shown.

As expected with our colony of NOD mice, the cumulative incidence of diabetes (as determined by two separate blood glucose concentrations ≥14 mmol/L) in the PBS-treated NOD mice reached a maximum at 30 weeks of age, with approximately 80% of animals developing diabetes and only 19% of animals remaining normoglycaemic (n = 11; Fig. 1A). In contrast, 84% of FhES-treated mice remained disease free at 30 weeks of age (experimental endpoint; n = 12; Fig. 1A). This data is representative of three independent studies in which there were no significant differences in survival rates between trials. In the other two trials, 88% (n = 16) and 86% (n = 7) of FhES-treated mice remained disease-free at 23 and 25 weeks, respectively (experimental endpoints), as compared to 13% (n = 16) and 17% (n = 12) of PBS-treated mice, respectively. Consistent with the ability of FhES to prevent autoimmune diabetes, examination of H&E-stained sections of pancreas isolated from FhES-treated mice at various time-points revealed a consistent and significant (*p*≤0.001) reduction in islet inflammation, as compared to PBS-treated mice (Fig. 1B–E). Collectively, these data demonstrate that FhES induced a robust and reproducible disease protection against autoimmune diabetes development.

### Diabetes prevention is associated with the suppression of autoreactive immune responses

**Figure 2:**
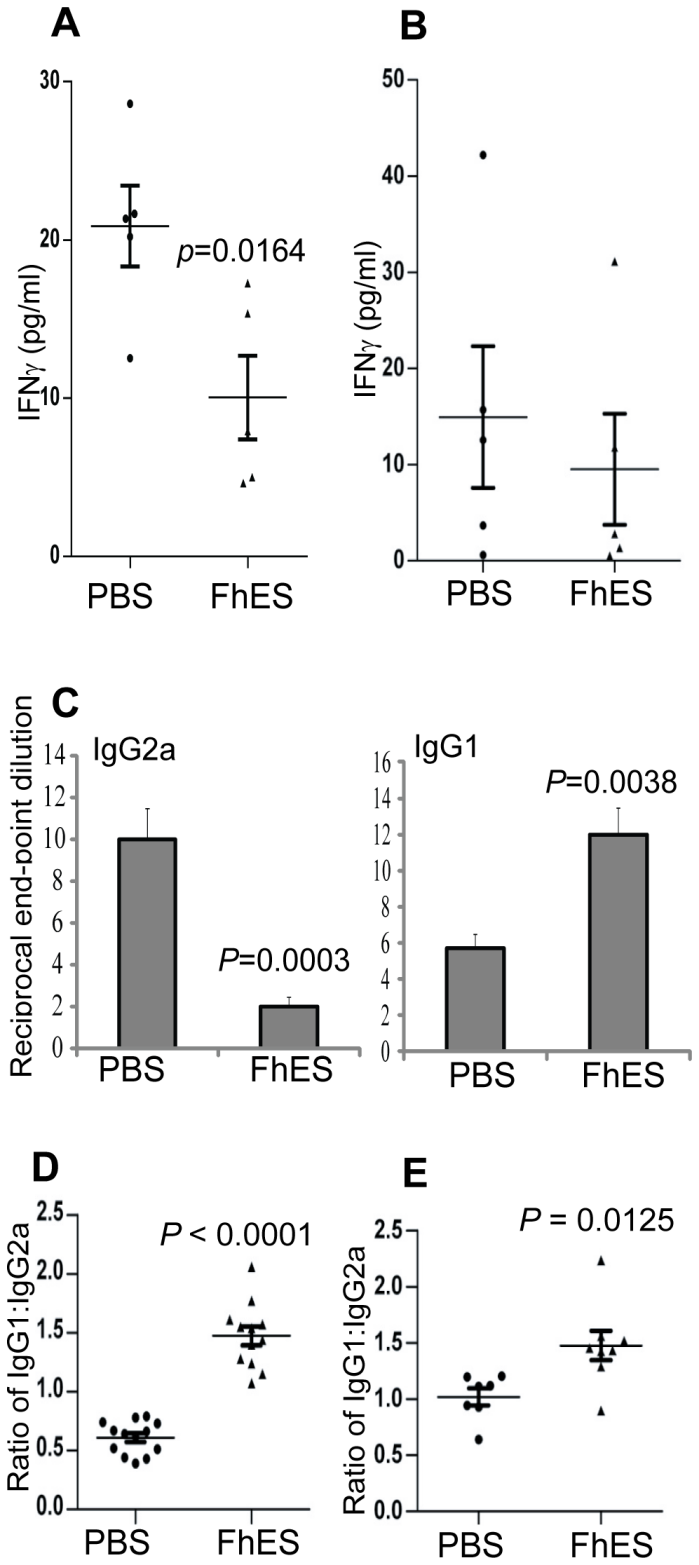
Treatment of NOD mice with FhES prevents the development of autoreactive immune responses. Female NOD mice (at four weeks of age; n = 5) were injected intraperitoneally with 10 µg of FhES (or PBS; vehicle control) on alternate days for a total of six injections. At (A) 24 h and (B) 7 weeks after the final injection spleen cells were isolated, stimulated with auto-antigen (insulin, 10 µg/ml) for 72 h and cell supernatants were assayed for IFN-γ. Data shown is representative of 3 independent experiments (C) Titres of insulin-specific IgG1 and IgG2a were measured in the sera of PBS- and FhES-treated mice (n = 15) at 15 weeks of age by ELISA. The data shown is representative of 3 independent experiments and displays the inverse of the end point serum dilution. (D) Ratios of IgG1 and IgG2a autoantibodies specific for insulin and (E) GAD in the sera of PBS- and FhES-treated mice (n = 15).

To examine if the disease protection in NOD mice afforded by FhES treatment was associated with modulation of autoantigen specific responses, we measured the amount of IFN-γ and IL-4 secreted from splenocytes *ex vivo* in response to stimulation with insulin (auto-antigen). The quantity of IFN-γ released from splenocytes, isolated from mice immediately after the final administration of FhES (6 weeks of age; Fig. 2A), were significantly (*p* = 0.0164) reduced, as compared to levels secreted by splenocytes from control (PBS treatment) mice. Even seven weeks after the final FhES treatment (at 13 weeks of age) when expansion of diabetogenic T cell clones would have normally already occurred, insulin-specific IFN-γ levels remained lower in the FhES-treated mice as compared to PBS-treated NOD mice (Fig. 2B). This suppression was specific to the development of antigen-specific responses as splenocytes isolated from FhES-treated mice secreted similar quantities of IFN-γ in response to stimulation with αCD3 (667±18 pg/ml), as compared to cells isolated from PBS-treated mice (646±7 pg/ml). In addition, the FhES mediated decrease in IFN-γ production was not due to a switch towards an antigen-specific Th2 response. At both 6 and 13 weeks of age, the levels of IL-4 secreted by splenocytes in response to insulin were below the level of detection (3 pg/ml) for both PBS- and FhES-treated mice. Cells isolated from both FhES- and PBS-treated mice secreted similar levels of IL-4 in response to stimulation with αCD3 (data not shown).

Since IFN-γ secretion by T cells stimulates the production of IgG2a [25], we isotyped autoreactive immunoglobulins in the sera of FhES- and PBS-treated mice. Specifically, we measured the titres of autoantibodies directed against the dominant auto-antigens, insulin and GAD. The levels of total IgG and IgM in sera from FhES-treated and PBS-treated mice did not differ (data not shown). However, treatment with FhES caused a switch towards the production of auto-antigen specific IgG1 (Fig. 2C), which resulted in a significant increase in the ratio of IgG1∶IgG2a autoantibodies against both insulin (Fig. 2D) and GAD (Fig. 2E) in mice treated with FhES, as compared to PBS-treatment. This data is consistent with the premise that diminished IFN-γ secretion, induced by FhES treatment, reduces the generation of auto-antigen-specific IgG2a responses from B cells.

### FhES treatment modulates immune cell populations in the pancreatic lymph nodes

In NOD mice, diabetogenic CD4^+^ T cells undergo priming and clonal expansion in the PLNs after presentation of islet autoantigens sequestered in the pancreas by antigen presenting cells (APCs) [26]. The immunological environment within the PLNs during the initiation phase of autoimmunity (co-incident with the delivery of FhES) is therefore a critical determinant for the expansion of autoreactive T cell clones. Accordingly, 24 h after the final treatment of FhES, we examined whether the treatment regime altered immune cell populations within the PLNs.

**Figure 3:**
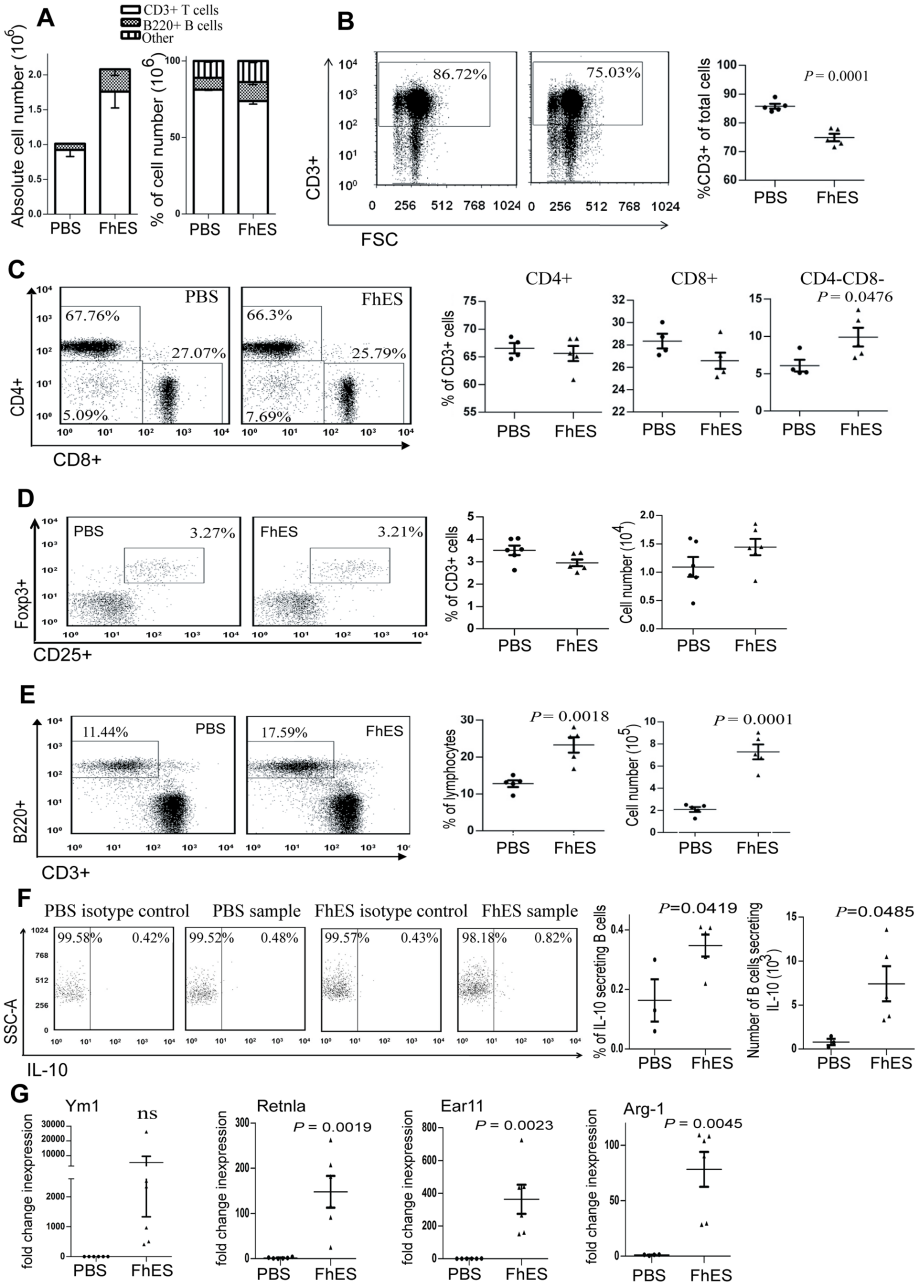
FhES treatment modulates the phenotype of immune cells in the pancreatic draining lymph nodes of NOD mice. Four-week old female NOD mice were treated with 10 µg of FhES or PBS intraperitoneally on alternate days for a total of six injections. The cellular composition within the PLNs was examined by flow cytometry 24 h after the final injection (n = 6; data representative of 5 independent experiments). (A) Absolute cell numbers and percentages of B220^+^ B cells and CD3^+^ T cells in the PLNs; (B) representative plots of the proportions of CD3^+^ T cells; (C) subsets of CD3^+^ T cells; (D) proportion and absolute numbers of CD4^+^CD25^+^Foxp3^+^ CD3^+^ T cells; (E) B220^+^ B cells; (F) representative dot plots of proportions (left panel), and absolute numbers (right panel) of IL-10 secreting CD19^+^ B cells within the CD19^+^ gate; (G) expression of Ym1, Retlna, Ear 11 and Arg-1 by quantitative realtime RT-PCR presented as fold change in expression, calculated compared to the average expression of the PBS cohort (each data point represents a single mouse; n = 6; data representative of at least 2 independent experiments).

While FhES treatment induced an increase in total numbers of immune cells within the PLNs (Fig. 3A), the proportion of CD3^+^ lymphocytes was significantly decreased (*p* = 0.0001; Fig. 3A&B). Within this T cell population, the proportion of CD8^+^ T cells and CD4^+^ T cells among FhES-treated and control mice remained similar, however the percentage of double negative (CD4^−^CD8^−^) T cells was elevated (*p* = 0.0476; Fig. 3C). Neither the proportion nor number of CD4^+^CD25^+^Foxp3^+^ Tregs differed significantly between FhES- and PBS- treatment groups (Fig. 3D).

A significant increase in both the proportion and absolute numbers of B220^+^ B cells in the PLNs of FhES-treated mice, as compared to PBS-treated mice, was observed (Fig. 3E). Given that infection with helminth parasites is associated with the generation of regulatory IL-10 secreting Bregs [27], we further characterised this expanded B cell population. The proportion of B cells within the PLNs that secreted IL-10 doubled following FhES treatment, which represented an overall 9.2-fold increase in the actual number of IL-10 secreting B cells in the PLNs compared to PBS-treated mice (Fig. 3F).

We were unable to detect a putative population of macrophages within the PLNs by flow cytometry. However, microarray analysis of PLNs showed that the highest increases in gene expression, following FhES-treatment, were for the characteristic markers of M2 macrophages (Ym1, Ear 11, Retnla and Arg1; data not shown). This data was validated by real-time quantitative RT-PCR, which confirmed a significant increase in the expression of these genes in the PLNs of FhES-treated mice compared to PBS-treated mice (Fig. 3G). The expression of these M2 markers was not detected in B or T cell populations that were purified by FACS (data not shown), suggesting the likely presence of a population of regulatory M2 macrophages within the PLNs of FhES-treated mice.

### FhES treatment activates M2 macrophages in the peritoneum

**Figure 4:**
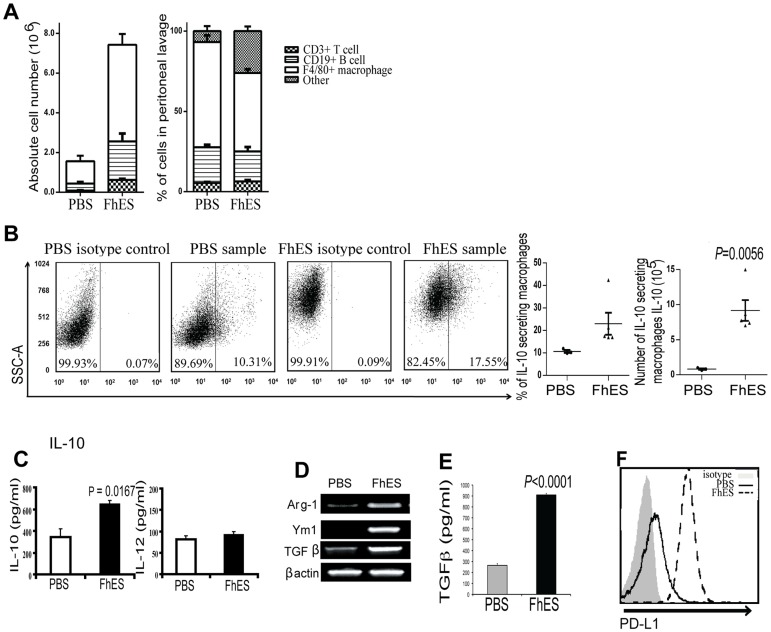
FhES treatment of NOD mice alters the populations of immune cells in the peritoneal cavity. Four-week old female NOD mice (n = 6) were treated with 10 µg of FhES or PBS intraperitoneally on alternate days for a total of six injections. The cellular composition and phenotype of cells within the peritoneal lavage fluid was examined 24 h after the final injection. (A) Absolute numbers and proportions of CD3^+^ T cells, CD19^+^ B cells and F4/80^+^ macrophages were quantified by flow cytometry; (B) representative dot plots of proportions (left panels) and absolute numbers (right panels) of IL-10 secreting F4/80^+^ macrophages within the F4/80^+^ gate; (C) spontaneous secretion of IL-10 and IL-12 by macrophages *ex vivo*; (D) expression of Ym1, Arg-1, and TGFβ in macrophages as determined by RT-PCR; (E) spontaneous secretion of TGFβ by macrophages *ex vivo*; and (F) Expression of PD-L1 on purified CD11b^+^ macrophages as analysed by flow cytometry. These data are representative of at least three independent experiments.

Considering that cells and antigens preferentially home to the PLNs and pancreas after intraperitoneal injection [28], [29], we postulated that the parasite-induced alterations to PLN immune cell populations were most likely initiated at the site of injection. Analyses of cellular populations following the final injection of FhES (at 6 weeks of age) showed that the total number of cells within the peritoneal lavage increased 6 fold compared to PBS treatment (data not shown). Similar to the PLNs, this rise in cell number was mainly attributable to an increase in the absolute numbers of macrophages and B cells (Figure 4A).

While there was no increase in the proportion of peritoneal B cells secreting IL-10 (data not shown), a significantly higher proportion of peritoneal macrophages from FhES-treated mice secreted IL-10 when compared to peritoneal macrophages isolated from PBS-treated mice (23.01% versus 10.68%, respectively; Fig. 4B). This macrophage population in FhES-treated mice represented 13.53% of the total numbers of cells and an 11.1-fold increase in the number of IL-10 secreting macrophages (Fig. 4B). Consistent with this data, an overall increase in the levels of IL-10 secreted *ex vivo* by peritoneal macrophages isolated from FhES-treated mice was observed compared to those isolated from PBS-treated animals (Fig. 4C). By contrast, no differences in the levels of IL-12 secreted from peritoneal macrophages isolated from FhES-treated and control mice were found (Fig. 4C).

Since secretion of IL-10 from macrophages is indicative of the activation of a regulatory M2 macrophage phenotype, we examined if FhES treatment also enhanced the expression levels of the other characteristic M2 markers, namely Ym1, Arg-1 and TGFβ. All of these M2 markers were expressed by macrophages isolated from FhES-treated mice, while negligible expression levels were detected in macrophages isolated from PBS-treated mice (Fig. 4D). In addition, peritoneal macrophages isolated from FhES-treated mice secreted significantly higher levels of bioactive TGFβ *ex vivo* compared to macrophages from PBS-treated animals (Fig. 4E). Furthermore, the surface expression levels of PD-L1 was increased on macrophages isolated from FhES-treated mice, as compared to PBS-treatment (Fig. 4F)

### FhES-activated M2 macrophages induce the expansion and/or survival of Foxp3^+^ regulatory T cells

**Figure 5:**
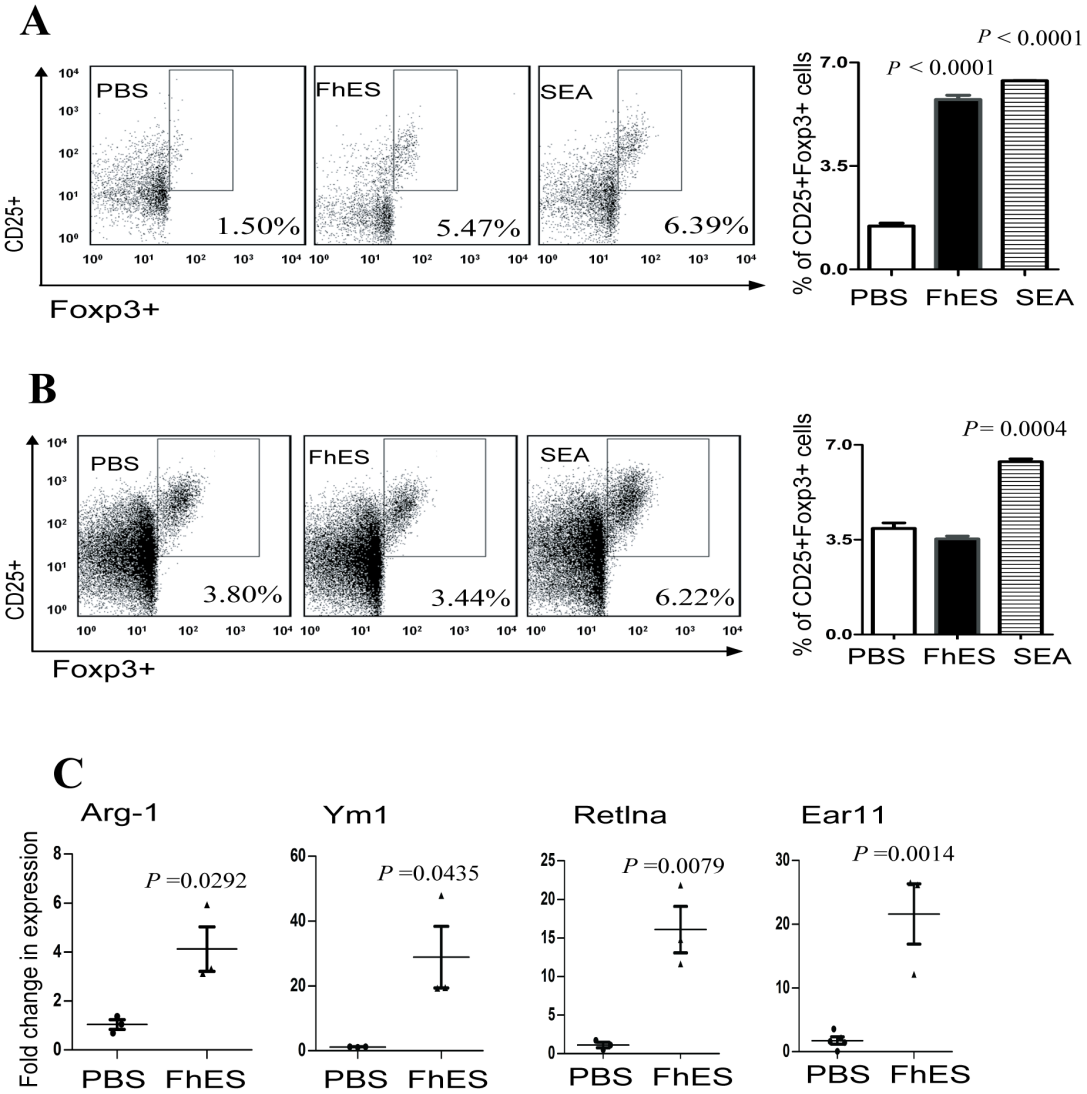
Peritoneal FhES-induced regulatory M2 macrophages expand Foxp3^+^ regulatory T cells *ex vivo*. (A) Four-week old female NOD mice were treated with 10 µg of FhES, SEA or PBS intraperitoneally on alternate days for a total of six injections. Peritoneal macrophages were harvested 24 h after the final injection and co-cultured with splenocytes isolated from age matched naive NOD mice in the presence of anti-CD3 (2 µg/ml) for 72 h. (B) Splenocytes were co-incubated with FhES (20 µg/ml), SEA (50 µg/ml), or PBS in the presence of anti-CD3 antibody (2 µg/ml) for 72 h. Representative flow cytometry dot plots are shown with the numbers representing the percentage of cells expressing both CD25^+^ and Foxp3. Histograms show the means of triplicate samples ± SEM, and are representative of two independent experiments. (C) Pancreata were isolated from female NOD mice (n = 3) 24 h after the final (sixth) FhES or PBS treatment and the expression levels of Arg1, Ym1, Retlna and Ear 11 were determined by quantitative realtime RT-PCR. All fold changes in expression levels were calculated compared to the average expression levels of the PBS cohort. Data shown is representative of at least two repeat experiments.

Given that the increase of M2 macrophages at the site of FhES injection and within the PLNs was the most pronounced effect of FhES-treatment, we next considered their potential function in mediating the prevention of T1D in NOD mice. The ability of M2 macrophages to influence the development of adaptive immune responses occurs via several mechanisms: (i) prevention of T cell proliferation [30], (ii) stimulation of the differentiation of antigen-specific Th2 cells [22], [31] or (iii) induction of the development of Tregs [32]. Our data indicated that neither T cell proliferation was affected by FhES-treatment nor were autoantigen-specific responses polarised towards a Th2 phenotype. Therefore, we investigated whether FhES-induced M2 macrophage populations modulated the development of Tregs. Thus, female NOD mice (at 4 weeks of age) were injected intraperitoneally with FhES (10 µg) on alternate days for a total of 6 injections and 24 hours after the final injection peritoneal macrophages were isolated and co-cultured with naive splenocytes *in vitro*. Co-incubation of FhES-derived macrophages led to a significant increase in the percentage of Foxp3^+^ Tregs that expanded in culture (Fig. 5A).

Zaccone *et al.* [33], [34] previously showed that, like FhES, peritoneal administration of SEA of the related helminth parasite *S. mansoni* protected NOD mice from T1D. Disease protection was associated with increased numbers of CD25^+^Foxp3^+^ Tregs in the pancreas of SEA-treated mice [34]. We, and others, have previously shown that, like FhES, peritoneal injection of SEA, induced the conversion of peritoneal macrophages to an M2 phenotype [22], [35]. Based on our observations of FhES modulation of macrophage activity we investigated whether macrophages isolated from SEA-treated NOD mice would behave analogously to those from FhES-treated mice.

Using the same treatment regime as described for FhES, female NOD mice (at 4 weeks of age) were injected intraperitoneally with SEA. Co-culture of peritoneal macrophages from these mice with naive splenocytes *in vitro* induced the same level of expansion of Foxp3^+^ Tregs to that observed after co-incubation of FhES-derived macrophages with splenocytes (Fig. 5A). The addition of FhES directly to naive splenocytes did not induce the expression of Foxp3 (Fig. 5B), further attributing this outcome to the presence of FhES-elicited M2 macrophages. By contrast, co-incubation of SEA with splenocytes *in vitro* led to a significant increase in the percentage of Tregs (Fig. 5B). This is consistent with previous reports suggesting that SEA expanded and/or induced the survival of Tregs via the induction of TGFβ secretion from DCs (34).

Expression levels of the M2 markers, Arg1, Ym1, Retlna, and Ear 11, were significantly increased in the pancreas of FhES-treated mice compared to controls (Fig. 5C respectively). This would suggest that FhES mediates protection in the NOD mouse via the induction of M2 macrophages, which in turn expand populations of Tregs and/or promote their survival.

## Discussion

Helminth parasites exert immune-modulatory effects in their hosts that prevent and/or attenuate auto-inflammatory diseases, such as multiple sclerosis, Crohn's disease and T1D [7]–[9]. The induction of Tregs and the associated secretion of IL-10 and TGFβ are events central to the immune responses induced during helminth infection and are also believed to be the principal mechanisms by which helminth parasites modulate autoimmune responses [11], [18], [34], [36]. In the present study, we show that the administration of molecules excreted/secreted by the helminth parasite *F. hepatica* (FhES) to NOD mice, at a time co-incident with T cell priming events, inhibits the initiation and perpetuation of autoimmune sequalae to prevent T1D. This finding is in agreement with previous reports showing that administration of the soluble homogenate of egg antigens from *S. mansoni* to NOD mice prevented the development of T1D [33], [34].

In NOD mice, diabetogenic CD4^+^ T cells undergo priming and clonal expansion in the PLNs after presentation of islet autoantigens sequestered in the pancreas by APCs [26]. Antigen availability and the cytokine environment within the PLNs during the initiation phase of autoimmunity (approximately 4 weeks of age) are critical determinants for the expansion of autoreactive T cell clones. When NOD mice were treated with FhES we observed an expansion of B cell populations within the PLNs, among which a subpopulation of putatively disease protective CD19^+^ IL-10 secreting B cells was increased. These IL-10-secreting B cells are characteristic of functional Bregs, which are activated during parasite infection [27], [37] and are thought to play an important role in controlling inflammation and pathology associated with *S. mansoni* infection. Although the specific function of this Breg population is yet to be elucidated, they can suppress antigen presentation [38] and promote anti-inflammatory Th2 immune responses [37]. Activated B cells also prevent autoimmune diabetes development in NOD mice, via an IL-10 dependent mechanism [39].

In the initiation stage of pathogenesis in NOD mice, classically-activated M1 macrophages secrete IL-12 to enhance the priming of diabetogenic cytotoxic T cells by DCs and B cells [40], [41]. In contrast, we found that M2 macrophages were expanded within the PLNs of mice treated with FhES. The secretion of IL-10 by these cells antagonises the activity of IL-12 secreting M1 macrophages, and, together with Breg populations in the PLNs, likely prevents the development of autoreactive Th1 immune responses. FhES-treated NOD mice also exhibited significantly increased numbers of regulatory M2 macrophages in the peritoneum and pancreas compared to PBS-treated mice and these populations were still evident at 15 weeks of age (9 weeks after FhES treatment; data not shown). This finding shows that a short-term treatment regime with FhES is sufficient to induce sustained immune-modulatory effects on the priming/expansion of diabetogenic T cell populations. Consequently, the majority of FhES-treated NOD mice remained normoglycaemic and had significantly reduced insulitic lesions, even at 30 weeks of age (experimental endpoint).

Previous studies have demonstrated that pancreatic M2 macrophages can prevent the development of T1D. For example, approximately 20% of female NOD mice never develop T1D and these animals exhibit increased numbers of regulatory M2 macrophages [42]. Calderon *et al.* [43] showed that transgenic NOD mice, which do not spontaneously develop T1D, express M2-associated genes within pancreatic islets. Recently, Parsa *et al.* [44] showed that adoptive transfer of M2 macrophages to 16 week-old pre-diabetic NOD mice protected 83% from TID for up to 3 months after treatment, at which time the mice were aged approximately 28 weeks. These results are comparable to the current study in which FhES prevented diabetes development in NOD mice for up to 30 weeks of age via a mechanism likely involving the induction of M2 macrophages.

It is plausible that FhES-stimulated M2 macrophages generated at the site of injection migrate to the pancreas. Indeed, pancreas isolated from NOD mice that had received an intraperitoneal injection of M2 macrophages derived from FhES-treated mice showed increased expression levels of Ym1 compared to mice that received macrophages isolated from PBS-treated mice (data not shown). A similar scenario has been reported for M2 macrophages adoptively transferred to NOD mice [44] and for M2 macrophages activated *in vivo* by the intraperitoneal injection of zymosan [45]. M2 macrophages have an enhanced phagocytic ability, which in the pancreas could accelerate clearance of apoptotic beta cells and thus prevent the initiation of beta cell-specific T cell responses [44]. However, M2 macrophages are not only directly protective against β-cell destruction but also indirectly through the expansion of CD4^+^CD25^+^Foxp3^+^ Tregs induced by TGFβ secretion. Here and elsewhere [21], [22], it was shown that macrophages stimulated with FhES *in vivo* and *in vitro* secrete TGFβ. Inhibition of autoantigen-specific Th1 and Th17 responses in a murine model of experimental allergic encephalomyelitis by infection with *F. hepatica* was also mediated by TGFβ [46]. In this case, a high proportion of both macrophages and Tregs produced TGFβ. However, although the authors proposed that Tregs mediated the protective effect, the role of TGFβ-secreting macrophages was not explored. Therefore, notwithstanding the fact that Tregs can afford protection from autoimmune disease, as has been demonstrated in a multitude of adoptive transfer studies, we suggest that during *F. hepatica* infection the modulation of macrophage function into a phenotype that acts to regulate immune responses (the expansion of Tregs being one such regulatory mechanism), may be the critical first step in the prevention of autoreactive immune responses.

Because an increased proportion of Tregs was observed in the pancreas of SEA-treated NOD mice, Zaccone *et al.* [34] suggested that CD25+CD4^+^Foxp3^+^ Tregs mediated protection from T1D. This hypothesis was supported by experiments showing that disease could be transferred to immunodeficient NOD.*scid* recipients using splenocytes isolated from SEA-treated NOD mice, which were depleted of CD25^+^CD4^+^Foxp3^+^ T cell populations, but not by non-depleted splenocytes. Despite the significant increase in M2 macrophage numbers in SEA-treated mice, a role for these cells in disease protection was not inferred [34], [35]. Here, we have shown that, similar to FhES, intraperitoneal administration of SEA to NOD mice induced M2 macrophages that are capable of expanding Foxp3^+^ Treg populations. Therefore, we suggest that these cells may also be pivotal in the schistosome-mediated protection against T1D in NOD mice.

It is now our interest to identify the individual molecules within FhES that induce the expansion of CD4^+^CD25^+^Foxp3 Tregs via the activation of M2-TGFβ secreting macrophages. Proteomics analysis revealed that FhES is a much less complex mix than SEA [47], [48]. Interestingly, the two major immune-modulatory molecules found in SEA, IPSE/α-1 [49] and glycoprotein ω-1 [50], are not found in FhES (although the carbohydrate moieties in FhES have yet to be characterised). Glycoprotein ω-1 induced Foxp3^+^ T cells from naive NOD CD4^+^ T cells *in vitro* via a mechanism dependent upon the secretion of TGFβ from DCs [51], although the therapeutic potential of this glycoprotein in NOD mice is yet to be established. By contrast, IPSE/α-1 did not induce the expansion of Foxp3^+^ Tregs. While FhES cannot directly induce Foxp3 expression in naive T cells, we have previously shown that both FhES and SEA contain a secreted molecule, peroxiredoxin (Prx), capable of activating macrophages to switch to an M2 phenotype, both *in vivo* and *in vitro* [21], [22].

We are currently investigating whether the induction of M2-TGFβ secreting macrophages by FhPrx and SmPrx in NOD mice is sufficient to mimic the protection afforded by FhES and SEA, respectively. Additionally, we are also investigating other molecules in FhES that influence macrophage function, such as cathepsin L protease [52] and cathelicidin-like helminth defense molecule (FhHDM) [53], which may work individually or synergistically. Most significantly, our data adds credence to the current thinking of exploiting isolated parasite molecules in the therapeutic treatment of autoimmune diseases in humans, including T1D.

## Supporting Information

Figure S1 **Facs Gating Strategy.** (A) Representative forward and side scatter gating strategy for the identification of lymphocytes within a single cell suspension of pancreatic lymph nodes; (B) representative forward and side scatter gating strategy for the identification of lymphocytes (G2) and monocytes (G3) within the total PEC; (C) representative gating strategy for single cells; and (D) representative gating strategy for CD19^+^ B cells, CD3^+^ T cells and F4/80^+^ macrophages within the PEC.
(TIF)
