# Supplementary figures and images for: Secreted Proteins from the Helminth Fasciola hepatica Inhibit the Initiation of Autoreactive T Cell Responses and Prevent Diabetes in the NOD Mouse
Source: PLoS One. 2014 Jan 21;9(1):e86289. doi: 10.1371/journal.pone.0086289 (PMC3897667; doi:10.1371/journal.pone.0086289)

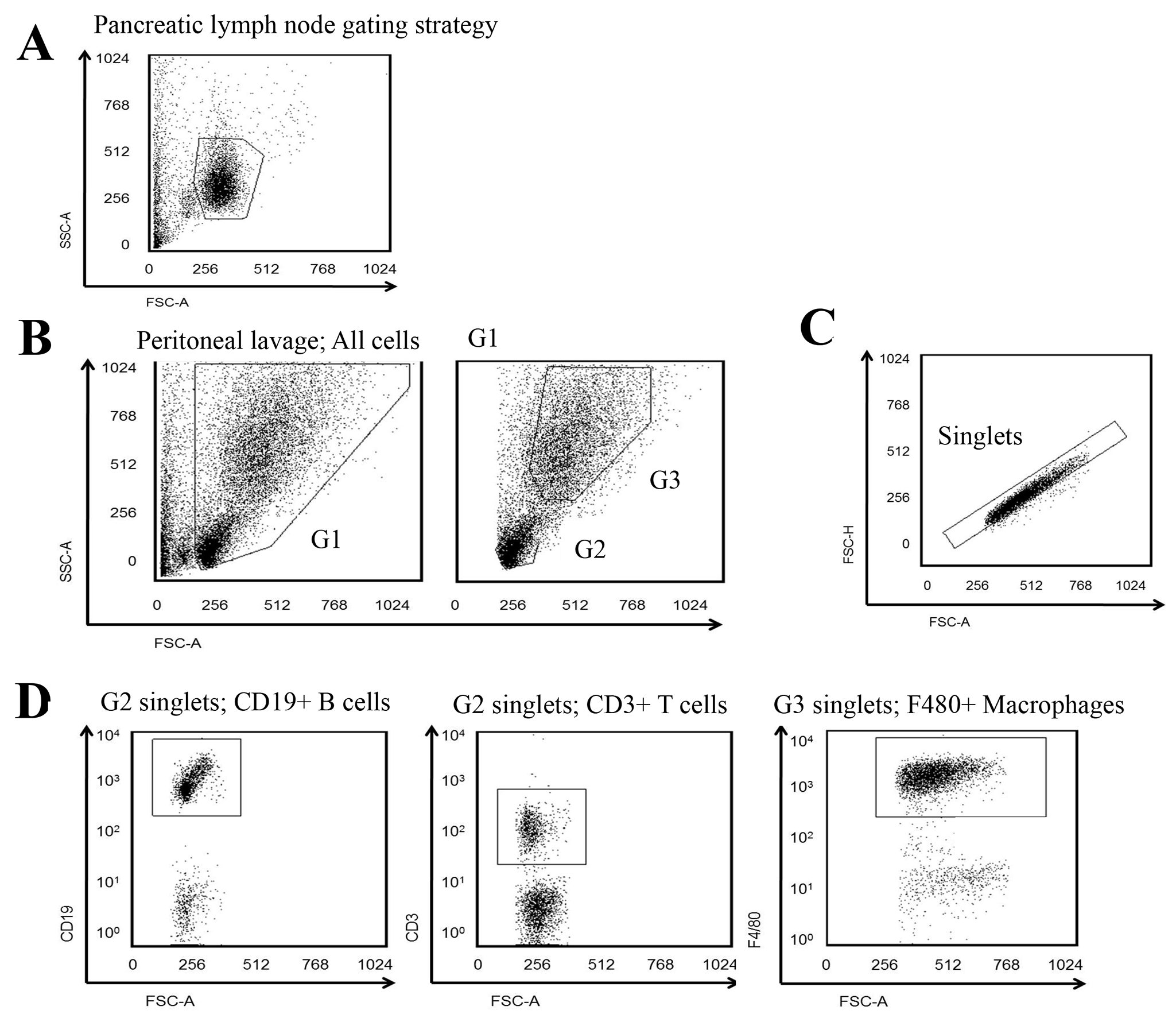

Supplement: Figure S1 — Facs Gating Strategy. (A) Representative forward and side scatter gating strategy for the identification of lymphocytes within a single cell suspension of pancreatic lymph nodes; (B) representative forward and side scatter gating strategy for the identification of lymphocytes (G2) and monocytes (G3) within the total PEC; (C) representative gating strategy for single cells; and (D) representative gating strategy for CD19+ B cells, CD3+ T cells and F4/80+ macrophages within the PEC. (TIF) [file pone.0086289.s001.tif]
